# Regulation of plant immune receptors by ubiquitination

**DOI:** 10.3389/fpls.2012.00238

**Published:** 2012-10-24

**Authors:** Giulia Furlan, Jörn Klinkenberg, Marco Trujillo

**Affiliations:** Leibniz Institute of Plant BiochemistryHalle (Saale), Germany

**Keywords:** E3 ubiquitin ligases, vesicle trafficking, receptor-like kinases, effectors, protein degradation, ubiquitination, PTI

## Abstract

From pathogen perception and the activation of signal transduction cascades to the deployment of defense responses, protein ubiquitination plays a key role in the modulation of plant immunity. Ubiquitination is mediated by three enzymes, of which the E3 ubiquitin ligases, the substrate determinants, have been the major focus of attention. Accumulating evidence suggests that ubiquitination modulates signaling mediated by pattern recognition receptors and is important for the accumulation of nucleotide-binding leucine-rich repeat type intracellular immune sensors. Recent studies also indicate that ubiquitination directs vesicle trafficking, a function that has been clearly established for immune signaling in animals. In this mini review, we discuss these and other recent advances and highlight important open questions.

## INTRODUCTION

Ubiquitin is a highlyrn Klinkenberg conserved protein found in all eukaryotes and is involved in almost all aspects of plant physiology, including immunity. Ubiquitination is the reversible attachment of ubiquitin moieties to specific target proteins and it is mediated by three enzymes (Vierstra, 2009). In the initial step, ubiquitin is activated by an ubiquitin-activating enzyme (E1). The activated ubiquitin is then transferred to an E2 ubiquitin-conjugating enzyme. A ubiquitin ligase (E3) then binds the E2 and the target protein. The ligase generally acts as a scaffold bringing the E2 and the target into close proximity to mediate the linkage of ubiquitin via its C-terminal glycine to an ε-Lysine (Lys) residue of the target. Because E3 ligases determine the specificity of the reaction they have attracted by far the most attention. Target proteins can be modified by the attachment of single ubiquitin molecules (monoubiquitination) or of ubiquitin polymers linked internally through one of seven Lys residues present in ubiquitin (polyubiquitination). Generally, conjugated ubiquitin monomers or polymers act as portable recognition modules that facilitate protein–protein interaction.

Lys48-linked ubiquitin chains promote protein breakdown by the 26S proteasome, a proteolytic complex that degrades the target with the concomitant release of the ubiquitin moieties for reuse. Alternatively linked ubiquitin chains can direct non-proteolytic events that participate in the regulation of vesicular trafficking, chromatin structure, and transcription (Ikeda and Dikic, 2008).

Post-translational modifications, such as ubiquitination, play key roles in signal transduction cascades. Understanding how such modifications translate into signal modulation has become a major research focus in recent years. This mini review focuses on recent reports implicating ubiquitination in the regulation of plant immune sensors and vesicle trafficking.

## UBIQUITINATION AND PATHOGEN PERCEPTION

The plant immune system can be conceptually divided into two branches characterized by different types of receptors (Jones and Dangl, 2006). The first branch is mediated by plasma membrane (PM) located pattern recognition receptors (PRRs), which recognize conserved pathogen molecules, so called pathogen-associated molecular patterns (PAMPs). Recognition of PAMPs by PRRs ultimately results in PAMP-triggered immunity (PTI). The second branch is activated by intracellular nucleotide-binding leucine-rich repeat (NB-LRR) immune sensors, which directly or indirectly perceive virulence factors, known as effectors, and results in the activation of effector-triggered immunity (ETI; Bent and Mackey, 2007).

A connection between ubiquitination and plant immunity was first suggested by a study showing that *suppressor of G2 allele of skp1* (*sgt1*) mutants were compromised in ETI (Azevedo et al., 2002). SGT1 is a component of the RAR1 (required for MLA12 resistance 1)-SGT1-HSP90 (heat shock protein 90) chaperone complex and association with components involved in protein ubiquitination has been shown for members of this complex. For example, S-phase kinase-associated protein 1 (SKP1) and its associated protein Cullin1, which are subunits of SKP1-Cullin1-F-box (SCF) ubiquitin ligases, were found to interact with SGT1 in plants (Azevedo et al., 2002; Liu et al., 2002). Subsequent studies have demonstrated that this chaperone complex plays a central role in the accumulation of NB-LRR proteins (Shirasu, 2009).

More recent findings have shown the direct regulation of NB-LRR accumulation through ubiquitin-mediated degradation via the 26S proteasome. Loss-of-function mutation of *constitutive expressor of PR genes 1 *(*CPR1*, also named *CPR30*), which encodes an F-box motif protein, leads to the accumulation of the Toll–interleukin-receptor-like (TIR) type NB-LRR protein SNC1 (suppressor of *npr1-1*, constitutive 1) and the coiled-coil (CC) type NB-LRR protein RPS2 (resistance to *Pseudomonas syringae* 2) resulting in autoimmune responses (Cheng et al., 2011; Gou et al., 2012). Accordingly, *CPR1* overexpression reduced SNC1 and RPS2 levels and immune response intensity. CPR1 was shown to interact with the ASK1 (*Arabidopsis* SKP1) and ASK2 subunits of SCF complexes. Furthermore, CPR1 interacts with SNC1 and RPS2, suggesting that they are its ubiquitination substrates and therefore mediate their stability (Gou et al., 2009; Cheng et al., 2011). Degradation mediated by CPR1 may reflect fine tuning mechanisms by which the plant is able to mount an immune response of appropriate intensity.

Another example of NB-LRR regulation by ubiquitination comes from a study conducted by Jeong et al. (2010), who uncovered an interesting link between light perception and immunity mediated by hypersensitive response to TCV (HRT), a CC-NB-LRR which mediates resistance against the *turnip crinkle virus* (TCV). HRT protein levels decreased in the dark or after blue-light induction, resulting in enhanced susceptibility. Application of proteasome inhibitor prevented blue-light-dependent degradation of HRT and consequently, plants were more resistant to TCV (Cooley et al., 2000; Jeong et al., 2010). HRT accumulation was reduced in mutants of the blue-light receptors cryptochrome 2 (CRY2) and phototropin 2 (PHO2). Importantly, HRT interacted with the ubiquitin ligase constitutively photomorphogenic 1 (COP1), but not with CRY2 or PHO2 (Jeong et al., 2010). Because CRY2 and PHO2 do interact with COP1 and they are required for HRT accumulation, it was proposed that they negatively regulate HRT degradation via COP1. However, the exact function of COP1 still remains to be determined.

**FIGURE 1:**
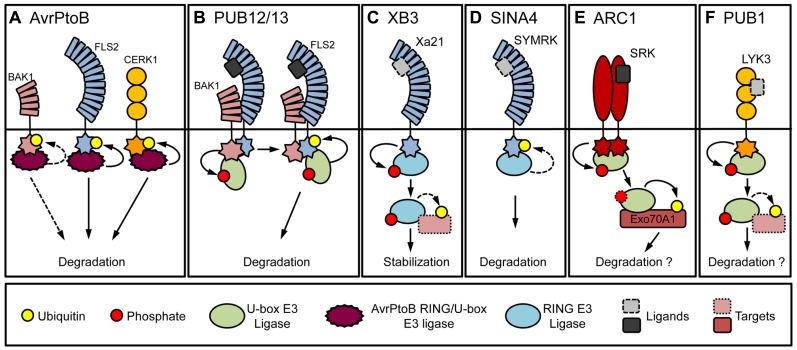
**Ubiquitin ligases that interact with receptor kinases**. **(A)** The effector protein AvrPtoB from *P. syringae* pv. *tomato* binds to the co-receptor BAK1, the LRR-RLK FLS2 and the LysM RLK CERK1. AvrPtoB is able to ubiquitinate FLS2 and CERK1 and mediate their degradation. AvrPtoB can ubiquitinate BAK1 weakly *in vitro*. The mechanism leading to reduced RLK levels by AvrPtoB activity *in vivo* requires further clarification. **(B)** PUB12 and PUB13 constitutively interact with the co-receptor kinase BAK1. Constitutive phosphorylation of PUB12 and PUB13 by BAK1 is enhanced by flg22 which induces the interaction with FLS2. PUB12 and PUB13 ubiquitinate FLS2 and mediate its degradation. **(C)** The rice XB3 ligase interacts with the LRR-RLK XA21. XA21 phosphorylates XB3 *in vitro*. Whether ligand binding is required for the phosphorylation is not known. XB3 contributes to XA21 accumulation and is therefore unlikely to ubiquitinate XA21. XB3 conceivably targets a protein that affects XA21 accumulation. **(D)** The *L. japonicus* SINA4 was shown to interact with and negatively regulate the levels of the LRR-RLK SYMRK, which mediates symbiotic signaling. **(E)** The *B. napus* ARC1 interacts and is phosphorylated by the S-domain SRK, which mediates SI reaction. ARC1 was proposed to regulate SI through the degradation of Exo70A1. Further experimental clarification is needed to determine whether ARC1 affects SRK levels. **(F)** The *M. truncatula* PUB1 interacts with and is phosphorylated by LYK3, a LysM type RLK involved in nodulation. PUB1, a negative regulator of nodulation, does not ubiquitinate LYK3 *in vitro*. PUB1 might therefore target an alternate protein required for symbiosis. Shapes with dotted lines denote potential involvement (e.g., ligand) or a hypothetical target.

In contrast to the intracellular NB-LRR immune sensors, surface-localized PRR receptor-like kinases (RLKs), relay external cues into the cell. PRRs recognize PAMPs such as flagellin, a component of the bacterial flagella, or chitin, a component of the fungal cell wall (Monaghan and Zipfel, 2012). Indication for the involvement of ubiquitination in the regulation of PRR signaling was first provided by the bacterial effector protein AvrPtoB, which is an active E3 ligase with a C-terminal U-box/RING-like domain (Janjusevic et al., 2006). AvrPtoB physically interacts with and ubiquitinates the flagellin receptor flagellin-sensitive 2 (FLS2; **Figure 1A)**. Expression of AvrPtoB resulted in a reduction of FLS2 levels, indicating that AvrPtoB facilitates its degradation (Göhre et al., 2008). AvrPtoB is also able to ubiquitinate and mediate the degradation of at least one more PRR, namely the chitin receptor chitin elicitor receptor kinase 1 (CERK1; Gimenez-Ibanez et al., 2009).

Recently, the plant U-box (PUB) ligases PUB12 and PUB13 were shown to mediate the endogenous ubiquitination of FLS2 (Lu et al., 2011; **Figure 1B**). PUB12 and PUB13 interact constitutively with brassinosteroid-insensitive 1-associated receptor kinase 1 (BAK1), while treatment with flg22, a conserved N-terminal peptide of flagellin, is required to induce their interaction with FLS2 (Chinchilla et al., 2007). Although phosphorylation of PUB12 and PUB13 by BAK1 was needed for the interaction with FLS2, it was dispensable for FLS2 ubiquitination. Interestingly, functional analysis of *pub12* and *pub13* mutants showed a phenotype reminiscent of *pub22*, *pub23*, and *pub24* mutants, which included enhanced responses to PAMPs and resistance to pathogens (Trujillo et al., 2008; Lu et al., 2011). Importantly, *pub12/pub13* double mutants displayed impaired reduction of FLS2 protein levels after flg22 treatment, indicating that they participate in the attenuation of signaling by regulating FLS2 turn-over. Of note, neither PUB12 or PUB13, nor AvrPtoB are able to effectively ubiquitinate BAK1 *in vitro* or affect BAK1 levels *in vivo*. Also, *in vitro* ubiquitination of FLS2 by PUB12, PUB13, and AvrPtoB is independent of its putative PEST domain although its mutation impairs endocytosis (Robatzek et al., 2006; Göhre et al., 2008; Lu et al., 2011). This suggests that FLS2 endocytosis and PUB12, PUB13, and AvrPtoB mediated degradation could be uncoupled.

PUB13 may have additional functions as suggested by studies which show that it negatively regulates cell death and influences flowering time (Li et al., 2012). *pub13* plants showed enhanced resistance against hemibiotrophic bacterial pathogens, in line with the results shown by Lu et al. (2011). Additionally, *pub13* mutants also displayed enhanced susceptibility to a necrotrophic pathogen (Li et al., 2012). Similarly, mutants of a gene encoding the putative PUB13 ortholog in rice, *spotted leaf 11 *(*SPL11*), were also reported to show spontaneous cell death and altered defense responses (Zeng et al., 2004). Interestingly, both orthologs additionally affect flowering time regulation, although they display opposing phenotypes. Whereas flowering starts earlier in *pub13* plants, it is delayed in rice *spl11* mutants grown under long day conditions (Vega-Sanchez et al., 2008; Li et al., 2012). Both resistance and flowering time phenotypes were shown to be largely dependent on constitutively increased SA levels in *pub13*, as introgression of *phytoalexin deficient 4* (*pad4*) or *salicylic acid induction deficient 2* (*sid2*) mutations suppressed both phenotypes.

In rice, the ubiquitin ligase XB3 (XA21-binding protein 3) interacts *in vivo* with the PRR XA21 (*Xanthomonas oryzae* pv. *oryzae* resistance 21), which is also able to phosphorylate XB3 (Wang et al., 2006). Reduced expression of *XB3* results in lower protein levels of XA21 and decreased resistance to the avirulent *X. oryzae *pv. *oryzae*, suggesting that XB3 is required for the accumulation of XA21 (**Figure 1C**). In *Lotus japonicus*, the RING-type ligase seven in absentia 4 (SINA4), was shown to interact with symbiosis RLK (SYMRK) and to negatively influence infection thread development during rhizobia infection (Den Herder et al., 2012; **Figure 1D**). Expression of SINA4 reduced SYMRK levels indicating a regulatory function of SINA4.

Because PRRs are integral membrane proteins, regulation of protein levels requires different cellular processes than in the case of NB-LRRs. Transport of RLKs to and from the PM is mediated by vesicle trafficking. Ubiquitination is closely involved in many steps of vesicle trafficking; it directs trafficking decisions related to both the biosynthetic secretory pathway and the removal of PM proteins via the endocytic pathway. Cell signaling and endocytic trafficking of membrane proteins have traditionally been regarded as two independent processes. However, recent studies, mainly from non-plant systems, have demonstrated that these two processes are intimately intertwined (Scita and Di Fiore, 2010).

## UBIQUITINATION AND IMMUNE RECEPTOR TRAFFICKING

Remodeling of the PM protein composition is emerging as a key aspect regulating receptor signaling and mediating signal resolution in space and time (Scita and Di Fiore, 2010). Endocytosis can regulate cell signaling by controlling the number of available receptors. This paradigm has been demonstrated for several receptors in animal cells including receptor tyrosine kinases, G protein-coupled receptors, and others (for review, see Sorkin and von Zastrow, 2009).

Recent studies suggest that a similar paradigm could be valid in plants. The receptor FLS2 is internalized and degraded in response to binding to flg22 (Robatzek et al., 2006). Internalization and concomitant degradation have been suggested to mediate signal attenuation.

The mechanism by which AvrPtoB-, PUB12-, or PUB13-mediated PRR ubiquitination modulates protein levels, still remains to be clarified. PRR ubiquitination can lead to one of many fates which can include endocytosis, changes in PRR sorting after endocytosis or in protein secretion. In humans, toll-like receptor (TLR)-mediated signaling is regulated by the RING-type ligase Triad3A. Both the TLR4 and TLR9 are ubiquitinated by Triad3A leading to their degradation upon activation with lipopolysaccharide (LPS) and cytosine-guanosine dinucleotide motifs (CpG), respectively (Chuang and Ulevitch, 2004). However, initial endocytosis of the LPS receptor complex can also take place in a ubiquitination-independent manner (Husebye et al., 2006). This suggests that receptor ubiquitination may regulate protein levels by modulating PRR traffic at different stages after endocytosis.

Following internalization, cargoes go through a sorting process which decides whether they will be recycled and returned to the PM, or transported to the vacuole for degradation via multi-vesicular bodies (MVBs). This additional level of regulation is mediated by the endosomal sorting complex required for transport (ESCRT). Several studies showed that monoubiquitination of integral PM proteins is required for sorting into MVBs in yeast and animal cells (Hicke, 2001; Haglund et al., 2003; Raiborg et al., 2003).

In plants, one of the first studies to show the involvement of ubiquitination in vacuolar sorting was provided by Kasai et al. (2011). They demonstrated that the substitution of the Lys590 residue, which is mono- or diubiquitinated *in vivo*, blocked the degradation of the borate transporter BOR1. Furthermore, the Lys590Ala mutation impaired translocation from the early endosome (EE) and transport to the vacuole without affecting localization to the PM. A recent study suggested a potential role of monoubiquitination in the degradation of the iron-regulated transporter 1 (IRT1), an integral PM protein, via the lytic vacuole (Barberon et al., 2011). IRT1 was shown to cycle between the PM, *trans*-Golgi network (TGN)/EE, and the vacuole to maintain optimal metal uptake. However, mutation of putative ubiquitin-conjugation residues led to IRT1 stabilization at the PM. In addition, artificial monoubiquitination of the PM ATPase was sufficient to cause its endocytosis and targeting to the vacuole, supporting monoubiquitination as signal for vacuolar targeting (Herberth et al., 2012). Because RLKs are integral membrane proteins, it is likely that they are also subject to similar processes in which ubiquitination orchestrates sorting into the vacuole.

Various components of the ESCRT bind ubiquitin and the deubiquitinating enzyme AMSH3 (associated molecule with the SH3 domain of STAM3) has been proposed to promote recycling of endocytosed proteins in animal cells. The *Arabidopsis* AMSH3 homolog is involved in vacuole biogenesis and vesicular traffic in general, including endocytosis (Isono et al., 2010). Interestingly, AMSH3 interacts with the ESCRT-III subunits vacuolar protein sorting 2.1 (VPS2.1) and VPS24.1 and regulates their localization (Katsiarimpa et al., 2011).

In the secretory pathway, components of the endoplasmic reticulum (ER)-quality control ensure the proper accumulation of PRRs. ER-quality control was shown to be required for the accumulation and proper function of elongation factor-Tu receptor (EFR) and FLS2 receptors (Nekrasov et al., 2009; Saijo et al., 2009). Mutant plants of the *stromal-derived factor-2 *(*SDF2*) and the *luminal binding protein *(*BiP*), a member of the Hsp70 family of chaperones, were impaired in PAMP-triggered responses and resistance against the pathogens *P. syringae* and *Alternaria brassicicola* (Nekrasov et al., 2009). The ER-quality control machinery is largely dependent on ubiquitination of defective proteins to mediate their degradation (Smith et al., 2011).

In addition, components of the ER-associated protein degradation (ERAD), such as the stress-induced ubiquitin-conjugating enzyme 32 (UBC32), participate in the secretion control of integral PM proteins. Transient expression of UBC32 in tobacco resulted in the reduced accumulation of the barley powdery mildew resistance locus O-12 (MLO12), a known substrate of ERAD (Müller et al., 2005; Cui et al., 2012). The *bri1-9* and *bri1-5* mutant alleles of the brassinosteroid-insensitive 1 (BRI1) receptor cause the ER-retention of the functional receptors and the typical brassinosteroid-insensitive dwarf phenotype (Jin et al., 2007; Hong et al., 2008). The double mutant *ubc32/bri1-9* partially rescues the *bri1-9* dwarf phenotype by allowing the functional bri1-9 mutant form to bypass ERAD and accumulate. Because UBC32 is induced by various ER stressors, it is conceivable that it participates in the regulation of ERAD stress responses (Cui et al., 2012). In line with these observations, two homologs of the ER membrane-localized RING-type ubiquitin ligase of the yeast and mammalian Hrd1, were shown to function redundantly in *bri1-9* ERAD (Su et al., 2010). Double mutants of the two *Arabidopsis Hrd1* homologs suppressed the *bri1-9* phenotype. The former observations are also interesting in light of recent data that show the antagonism between brassino-steroid and immune signaling (Albrecht et al., 2011; Belkhadir et al., 2011).

## REGULATION OF UBIQUITIN LIGASES

In many cases ubiquitin ligases are phosphorylated by interacting RLKs. It is therefore tempting to speculate that ligase phosphorylation regulates their activity or the interaction with target proteins.

One of the first examples showing such an interaction was the U-box type ubiquitin ligase from *Brassica napus* arm repeat containing 1 (ARC1) and S receptor kinase (SRK) which regulates self-incompatibility (SI; **Figure 1E;** Stone et al., 1999). ARC1 was shown to be phosphorylated by SRK (Gu et al., 1998). Interestingly, phosphorylation was required for the relocalization of ARC1 from the cytosol to the ER (Stone et al., 2003). Yeast two-hybrid analysis with different S-domain RLKs and several *Arabidopsis* PUBs suggested the conservation of the SI signaling pathway in *Arabidopsis* (Samuel et al., 2008). The *Arabidopsis* S-domain RLKs *Arabidopsis* receptor kinase 1 (ARK1) and ARK2 were also able to phosphorylate PUB9 and PUB13 *in vitro*. In addition, the *Nicotiana benthamiana* RLK CHRK1 had previously been reported to interact with *Nt*PUB4, the homolog of *Bn*ARC1 (Kim et al., 2003).

The *Medicago truncatula *PUB1 was shown to interact with lysin motif RLK 3 (LYK3), a putative RLK of *Sinorhizobium meliloti* Nod factors (Mbengue et al., 2010). PUB1 was also phosphorylated by LYK3, but was unable to ubiquitinate it *in vitro* (**Figure 1F**). Overexpression and knock-down experiments suggested that PUB1 is a negative regulator of infection and nodulation by *S. meliloti*.

In the case of PUB12 and PUB13, BAK1-mediated phosphorylation induced their association with FLS2 (Lu et al., 2011), suggesting that phosphorylation modulates ligase affinity and thus mediates the association to FLS2. However, PUB12 and PUB13 phosphorylation does not seem to be required for target ubiquitination, since they readily ubiquitinated FLS2 *in vitro*. Furthermore, most RING and PUB ligases display *in vitro* autoubiquitination, suggesting that additional factors are dispensable for their activity. Nevertheless, it still remains unknown whether PUB1, SINA4, or XB3, as well as other mentioned ubiquitin ligases, can ubiquitinate the corresponding RLKs. Instead, it is conceivable that phosphorylation triggers the interaction with alternative targets.

The relocalization of proteins prompted by interaction with ubiquitin ligases is a reoccurring theme. Intracellular relocalization of ubiquitin ligases may represent a mechanism by which their activity is restricted to a specific cellular context. The RING-type ligase keep on going (KEG) functions in abscisic acid signaling and its mutation suppresses the enhanced resistance against powdery mildew in *enhanced disease resistance 1 *(*edr1*) plants (Wawrzynska et al., 2008). KEG interacts with EDR1, which was shown to localize to the ER. EDR1 is recruited by KEG to the TGN/EE when coexpressed (Gu and Innes, 2011). Another example is the previously mentioned ARC1, shown to interact with Exo70A1, a subunit of the exocyst complex. Coexpression of Exo70A1 with ARC1 resulted in their relocalization from the cytosol to punctate structures (Samuel et al., 2009). Similarly, SYMRK relocalizes from the PM to punctate structures in the cytosol in the presence of SINA4 (Den Herder et al., 2012). However, whether target ubiquitination is required for the relocalization, still needs to be shown. Further work is necessary to resolve the dynamic interactions and modifications occurring between regulatory ligases and immune receptor kinases.

## CONCLUSIONS AND PERSPECTIVES

Surfacing data showing the manifold and central functions of ubiquitination in vesicle trafficking represent a preliminary confirmation in plants of long standing paradigms in yeast and animal cells. However, the general scarcity of ubiquitination targets still obstructs insight into the cellular processes that are being regulated. Furthermore, it is necessary to discriminate between the different types of ubiquitination, since these mediate distinct fates of the tagged proteins. The importance of this aspect becomes apparent if one considers that ubiquitin is a common denominator involved in targeting of substrates to all three major protein degradation pathways in mammalian cells: the proteasome, the lysosome, and the autophagosome. In plants, most attention has been focused on the role of ubiquitination in mediating the turn-over of modified proteins by the proteasome, while relatively little is known about its role in directing proteins into the vacuole or autophagocytosis. However, at this point, the major challenge continues to be the identification of ligase targets.

## Conflict of Interest Statement

The authors declare that the research was conducted in the absence of any commercial or financial relationships that could be construed as a potential conflict of interest.

## References

[B1] Albrecht C. Boutrot F. Segonzac C. Schwessinger B. Gimenez-Ibanez S. Chinchilla D. (2011). Brassinosteroids inhibit pathogen-associated molecular pattern-triggered immune signaling independent of the receptor kinase BAK1. *Proc. Natl. Acad. Sci. U.S.A.* 109 303–308 22087006 10.1073/pnas.1109921108 PMC3252947

[B2] Azevedo C. Sadanandom A. Kitagawa K. Freialdenhoven A. Shirasu K. Schulze-Lefert P. (2002). The RAR1 interactor SGT1, an essential component of R gene-triggered disease resistance. *Science* 295 2073–2076 11847307 10.1126/science.1067554

[B3] Barberon M. Zelazny E. Robert S. Conejero G. Curie C. Friml J. (2011). Monoubiquitin-dependent endocytosis of the iron-regulated transporter 1 (IRT1) transporter controls iron uptake in plants. *Proc. Natl. Acad. Sci. U.S.A.* 108 E450–E458 21628566 10.1073/pnas.1100659108 PMC3156158

[B4] Belkhadir Y. Jaillais Y. Epple P. Balsemao-Pires E. Dangl J. L. Chory J. (2011). Brassinosteroids modulate the efficiency of plant immune responses to microbe-associated molecular patterns. *Proc. Natl. Acad. Sci. U.S.A.* 109 297–302 22087001 10.1073/pnas.1112840108 PMC3252953

[B5] Bent A. F. Mackey D. (2007). Elicitors, effectors, and R genes: the new paradigm and a lifetime supply of questions. *Annu. Rev. Phytopathol.* 45 399–436 17506648 10.1146/annurev.phyto.45.062806.094427

[B6] Cheng Y. T. Li Y. Huang S. Huang Y. Dong X. Zhang Y. (2011). Stability of plant immune-receptor resistance proteins is controlled by SKP1-Cullin1-F-box (SCF)-mediated protein degradation. *Proc. Natl. Acad. Sci. U.S.A.* 108 14694–14699 21873230 10.1073/pnas.1105685108 PMC3167521

[B7] Chinchilla D. Zipfel C. Robatzek S. Kemmerling B. Nurnberger T. Jones J. D. (2007). A flagellin-induced complex of the receptor FLS2 and BAK1 initiates plant defence. *Nature* 448 497–500 17625569 10.1038/nature05999

[B8] Chuang T. H. Ulevitch R. J. (2004). Triad3A, an E3 ubiquitin-protein ligase regulating Toll-like receptors. *Nat. Immunol.* 5 495–502 15107846 10.1038/ni1066

[B9] Cooley M. B. Pathirana S. Wu H. J. Kachroo P. Klessig D. F. (2000). Members of the *Arabidopsis* HRT/RPP8 family of resistance genes confer resistance to both viral and oomycete pathogens. *Plant Cell* 12 663–676 10810142 10.1105/tpc.12.5.663 PMC139919

[B10] Cui F. Liu L. Zhao Q. Zhang Z. Li Q. Lin B. (2012). *Arabidopsis* ubiquitin conjugase UBC32 is an ERAD component that functions in brassinosteroid-mediated salt stress tolerance. *Plant Cell* 24 233–244 22214659 10.1105/tpc.111.093062 PMC3289556

[B11] Den Herder G. Yoshida S. Antolin-Llovera M. Ried M. K. Parniske M. (2012). *Lotus japonicus* E3 Ligase SEVEN IN ABSENTIA4 destabilizes the symbiosis receptor-like kinase SYMRK and negatively regulates rhizobial infection. *Plant Cell* 24 1691–1707 22534128 10.1105/tpc.110.082248 PMC3398572

[B12] Gimenez-Ibanez S. Hann D. R. Ntoukakis V. Petutschnig E. Lipka V. Rathjen J. P. (2009). AvrPtoB targets the LysM receptor kinase CERK1 to promote bacterial virulence on plants. *Curr. Biol.* 19 423–429 19249211 10.1016/j.cub.2009.01.054

[B13] Göhre V. Spallek T. Haweker H. Mersmann S. Mentzel T. Boller T. (2008). Plant pattern-recognition receptor FLS2 is directed for degradation by the bacterial ubiquitin ligase AvrPtoB. *Curr. Biol.* 18 1824–1832 19062288 10.1016/j.cub.2008.10.063

[B14] Gou M. Su N. Zheng J. Huai J. Wu G. Zhao J. (2009). An F-box gene, CPR30, functions as a negative regulator of the defense response in *Arabidopsis*. *Plant J.* 60 757–770 19682297 10.1111/j.1365-313X.2009.03995.x

[B15] Gou M. Shi Z. Zhu Y. Bao Z. Wang G. Hua J. (2012). The F-box protein CPR1/CPR30 negatively regulates R protein SNC1 accumulation. *Plant J.* 69 411–420 21967323 10.1111/j.1365-313X.2011.04799.x

[B16] Gu T. Mazzurco M. Sulaman W. Matias D. D. Goring D. R. (1998). Binding of an arm repeat protein to the kinase domain of the S-locus receptor kinase. *Proc. Natl. Acad. Sci. U.S.A.* 95 382–387 9419384 10.1073/pnas.95.1.382 PMC18231

[B17] Gu Y. Innes R. W. (2011). The KEEP ON GOING protein of *Arabidopsis* recruits the ENHANCED DISEASE RESISTANCE1 protein to *trans*-Golgi network/early endosome vesicles. *Plant Physiol.* 155 1827–1838 21343429 10.1104/pp.110.171785 PMC3091131

[B18] Haglund K. Sigismund S. Polo S. Szymkiewicz I. Di Fiore P. P. Dikic I. (2003). Multiple monoubiquitination of RTKs is sufficient for their endocytosis and degradation. *Nat. Cell. Biol.* 5 461–466 12717448 10.1038/ncb983

[B19] Herberth S. Shahriari M. Bruderek M. Hessner F. Muller B. Hulskamp M. (2012). Artificial ubiquitylation is sufficient for sorting of a plasma membrane ATPase to the vacuolar lumen of *Arabidopsis *cells. *Planta* 236 63–77 22258747 10.1007/s00425-012-1587-0

[B20] Hicke L. (2001). Protein regulation by monoubiquitin. *Nat. Rev. Mol. Cell. Biol.* 2 195–201 11265249 10.1038/35056583

[B21] Hong Z. Jin H. Tzfira T. Li J. (2008). Multiple mechanism-mediated retention of a defective brassinosteroid receptor in the endoplasmic reticulum of *Arabidopsis*. *Plant Cell* 20 3418–3429 19060110 10.1105/tpc.108.061879 PMC2630446

[B22] Husebye H. Halaas O. Stenmark H. Tunheim G. Sandanger O. Bogen B. (2006). Endocytic pathways regulate Toll-like receptor 4 signaling and link innate and adaptive immunity. *EMBO J.* 25 683–692 16467847 10.1038/sj.emboj.7600991 PMC1383569

[B23] Ikeda F. Dikic I. (2008). Atypical ubiquitin chains: new molecular signals. ‘Protein Modifications: Beyond the Usual Suspects’ review series. *EMBO Rep.* 9 536–542 18516089 10.1038/embor.2008.93 PMC2427391

[B24] Isono E. Katsiarimpa A. Muller I. K. Anzenberger F. Stierhof Y. D. Geldner N. (2010). The deubiquitinating enzyme AMSH3 is required for intracellular trafficking and vacuole biogenesis in *Arabidopsis thaliana*. *Plant Cell* 22 1826–1837 20543027 10.1105/tpc.110.075952 PMC2910964

[B25] Janjusevic R. Abramovitch R. B. Martin G. B. Stebbins C. E. (2006). A bacterial inhibitor of host programmed cell death defenses is an E3 ubiquitin ligase. *Science* 311 222–226 16373536 10.1126/science.1120131

[B26] Jeong R. D. Chandra-Shekara A. C. Barman S. R. Navarre D. Klessig D. F. Kachroo A. (2010). Cryptochrome 2 and phototropin 2 regulate resistance protein-mediated viral defense by negatively regulating an E3 ubiquitin ligase. *Proc. Natl. Acad. Sci. U.S.A.* 107 13538–13543 20624951 10.1073/pnas.1004529107 PMC2922132

[B27] Jin H. Yan Z. Nam K. H. Li J. (2007). Allele-specific suppression of a defective brassinosteroid receptor reveals a physiological role of UGGT in ER quality control. *Mol. Cell* 26 821–830 17588517 10.1016/j.molcel.2007.05.015 PMC1948852

[B28] Jones J. D. Dangl J. L. (2006). The plant immune system. *Nature* 444 323–329 17108957 10.1038/nature05286

[B29] Kasai K. Takano J. Miwa K. Toyoda A. Fujiwara T. (2011). High boron-induced ubiquitination regulates vacuolar sorting of the BOR1 borate transporter in *Arabidopsis thaliana*. *J. Biol. Chem.* 286 6175–6183 21148314 10.1074/jbc.M110.184929 PMC3057829

[B30] Katsiarimpa A. Anzenberger F. Schlager N. Neubert S. Hauser M. T. Schwechheimer C. (2011). The *Arabidopsis* deubiquitinating enzyme AMSH3 interacts with ESCRT-III subunits and regulates their localization. *Plant Cell* 23 3026–3040 21810997 10.1105/tpc.111.087254 PMC3180808

[B31] Kim M. Cho H. S. Kim D. M. Lee J. H. Pai H. S. (2003). CHRK1, a chitinase-related receptor-like kinase, interacts with NtPUB4, an armadillo repeat protein, in tobacco. *Biochim. Biophys. Acta* 1651 50–59 14499588 10.1016/s1570-9639(03)00234-6

[B32] Li W. Ahn I. P. Ning Y. Park C. H. Zeng L. Whitehill J. (2012). The U-box/ARM E3 ligase PUB13 regulates cell death, defense and flowering time in *Arabidopsis*. *Plant Physiol*. 159 239–250 22383540 10.1104/pp.111.192617 PMC3366716

[B33] Liu Y. Schiff M. Serino G. Deng X. W. Dinesh-Kumar S. P. (2002). Role of SCF ubiquitin-ligase and the COP9 signalosome in the N gene-mediated resistance response to Tobacco mosaic virus. *Plant Cell* 14 1483–1496 12119369 10.1105/tpc.002493 PMC150701

[B34] Lu D. Lin W. Gao X. Wu S. Cheng C. Avila J. (2011). Direct ubiquitination of pattern recognition receptor FLS2 attenuates plant innate immunity. *Science* 332 1439–1442 21680842 10.1126/science.1204903 PMC3243913

[B35] Mbengue M. Camut S. de Carvalho-Niebel F. Deslandes L. Froidure S. Klaus-Heisen D. (2010). The *Medicago truncatula* E3 ubiquitin ligase PUB1 interacts with the LYK3 symbiotic receptor and negatively regulates infection and nodulation. *Plant Cell* 22 3474–3488 20971894 10.1105/tpc.110.075861 PMC2990133

[B36] Monaghan J. Zipfel C. (2012). Plant pattern recognition receptor complexes at the plasma membrane. *Curr. Opin. Plant Biol.* 15 349–357 22705024 10.1016/j.pbi.2012.05.006

[B37] Müller J. Piffanelli P. Devoto A. Miklis M. Elliott C. Ortmann B. (2005). Conserved ERAD-like quality control of a plant polytopic membrane protein. *Plant Cell* 17 149–163 15598804 10.1105/tpc.104.026625 PMC544496

[B38] Nekrasov V. Li J. Batoux M. Roux M. Chu Z. H. Lacombe S. (2009). Control of the pattern-recognition receptor EFR by an ER protein complex in plant immunity. *EMBO J.* 28 3428–3438 19763086 10.1038/emboj.2009.262 PMC2776097

[B39] Raiborg C. Rusten T. E. Stenmark H. (2003). Protein sorting into multivesicular endosomes. *Curr. Opin. Cell Biol.* 15 446–455 12892785 10.1016/s0955-0674(03)00080-2

[B40] Robatzek S. Chinchilla D. Boller T. (2006). Ligand-induced endocytosis of the pattern recognition receptor FLS2 in *Arabidopsis*. *Genes Dev.* 20 537–542 16510871 10.1101/gad.366506 PMC1410809

[B41] Saijo Y. Tintor N. Lu X. Rauf P. Pajerowska-Mukhtar K. Haweker H. (2009). Receptor quality control in the endoplasmic reticulum for plant innate immunity. *EMBO J.* 28 3439–3449 19763087 10.1038/emboj.2009.263 PMC2776098

[B42] Samuel M. A. Mudgil Y. Salt J. N. Delmas F. Ramachandran S. Chilelli A. (2008). Interactions between the S-domain receptor kinases and AtPUB-ARM E3 ubiquitin ligases suggest a conserved signaling pathway in *Arabidopsis*. *Plant Physiol.* 147 2084–2095 18552232 10.1104/pp.108.123380 PMC2492606

[B43] Samuel M. A. Chong Y. T. Haasen K. E. Aldea-Brydges M. G. Stone S. L. Goring D. R. (2009). Cellular pathways regulating responses to compatible and self-incompatible pollen in *Brassica* and *Arabidopsis* stigmas intersect at Exo70A1, a putative component of the exocyst complex. *Plant Cell* 21 2655–2671 19789280 10.1105/tpc.109.069740 PMC2768929

[B44] Scita G Di Fiore P. P. (2010). The endocytic matrix. *Nature* 463 464–473 20110990 10.1038/nature08910

[B45] Shirasu K. (2009). The HSP90-SGT1 chaperone complex for NLR immune sensors. *Annu. Rev. Plant Biol.* 60 139–164 19014346 10.1146/annurev.arplant.59.032607.092906

[B46] Smith M. H. Ploegh H. L. Weissman J. S. (2011). Road to ruin: targeting proteins for degradation in the endoplasmic reticulum. *Science* 334 1086–1090 22116878 10.1126/science.1209235 PMC3864754

[B47] Sorkin A von Zastrow M. (2009). Endocytosis and signalling: intertwining molecular networks. *Nat. Rev. Mol. Cell Biol.* 10 609–622 19696798 10.1038/nrm2748 PMC2895425

[B48] Stone S. L. Anderson E. M. Mullen R. T. Goring D. R. (2003). ARC1 is an E3 ubiquitin ligase and promotes the ubiquitination of proteins during the rejection of self-incompatible *Brassica* pollen. *Plant Cell* 15 885–898 12671085 10.1105/tpc.009845 PMC152337

[B49] Stone S. L. Arnoldo M. Goring D. R. (1999). A breakdown of *Brassica* self-incompatibility in ARC1 antisense transgenic plants. *Science* 286 1729–1731 10576738 10.1126/science.286.5445.1729

[B50] Su W. Liu Y. Xia Y. Hong Z. Li J. (2010). Conserved endoplasmic reticulum-associated degradation system to eliminate mutated receptor-like kinases in *Arabidopsis*. *Proc. Natl. Acad. Sci. U.S.A.* 108 870–875 21187394 10.1073/pnas.1013251108 PMC3021050

[B51] Trujillo M. Ichimura K. Casais C. Shirasu K. (2008). Negative regulation of PAMP-triggered immunity by an E3 ubiquitin ligase triplet in *Arabidopsis*. *Curr. Biol.* 18 1396–1401 18771922 10.1016/j.cub.2008.07.085

[B52] Vega-Sanchez M. E. Zeng L. Chen S. Leung H. Wang G. L. (2008). SPIN1, a K homology domain protein negatively regulated and ubiquitinated by the E3 ubiquitin ligase SPL11, is involved in flowering time control in rice. *Plant Cell* 20 1456–1469 18586868 10.1105/tpc.108.058610 PMC2483366

[B53] Vierstra R. D. (2009). The ubiquitin-26S proteasome system at the nexus of plant biology. *Nat. Rev. Mol. Cell Biol.* 10 385–397 19424292 10.1038/nrm2688

[B54] Wang Y. S. Pi L. Y. Chen X. Chakrabarty P. K. Jiang J. De Leon A. L. (2006). Rice XA21 binding protein 3 is a ubiquitin ligase required for full Xa21-mediated disease resistance. *Plant Cell* 18 3635–3646 17172358 10.1105/tpc.106.046730 PMC1785399

[B55] Wawrzynska A. Christiansen K. M. Lan Y. Rodibaugh N. L. Innes R. W. (2008). Powdery mildew resistance conferred by loss of the ENHANCED DISEASE RESISTANCE1 protein kinase is suppressed by a missense mutation in KEEP ON GOING, a regulator of abscisic acid signaling. *Plant Physiol.* 148 1510–1522 18815384 10.1104/pp.108.127605 PMC2577273

[B56] Zeng L. R. Qu S. Bordeos A. Yang C. Baraoidan M. Yan H. (2004). Spotted leaf11, a negative regulator of plant cell death and defense, encodes a U-box/armadillo repeat protein endowed with E3 ubiquitin ligase activity. *Plant Cell* 16 2795–2808 15377756 10.1105/tpc.104.025171 PMC520972

